# Efficacy and Safety of Self-Expandable Metallic Stent Placement for Malignant Esophageal Fistula

**DOI:** 10.3390/jcm12185859

**Published:** 2023-09-08

**Authors:** Atsuko Izumi, Toshiyuki Yoshio, Takashi Sasaki, Mitsuaki Ishioka, Atsuko Kizawa, Yohei Ikenoyama, Ken Namikawa, Yoshitaka Tokai, Shoichi Yoshimizu, Yusuke Horiuchi, Akiyoshi Ishiyama, Toshiaki Hirasawa, Keisho Chin, Mariko Ogura, Naoki Sasahira, Junko Fujisaki

**Affiliations:** 1 Department of Gastroenterology, Cancer Institute Hospital, Japanese Foundation for Cancer Research, Tokyo 135-8550, Japan; atsuko.izumi@jfcr.or.jp (A.I.); mitsuaki.ishioka@jfcr.or.jp (M.I.); atsuko.tamashiro@oici.jp (A.K.); yohei.ikenoyama@jfcr.or.jp (Y.I.); ken.namikawa@jfcr.or.jp (K.N.); yoshitaka.tokai@jfcr.or.jp (Y.T.); shoichi.yoshimizu@jfcr.or.jp (S.Y.); yusuke.horiuchi@jfcr.or.jp (Y.H.); akiyoshi.ishiyama@jfcr.or.jp (A.I.); toshiaki.hirasawa@jfcr.or.jp (T.H.); junko.fujisaki@jfcr.or.jp (J.F.); 2 Department of Hepato-Biliary-Pancreatic Medicine, Cancer Institute Hospital, Japanese Foundation for Cancer Research, Tokyo 135-8550, Japan; takashi.sasaki@jfcr.or.jp (T.S.); naoki.sasahira@jfcr.or.jp (N.S.); 3 Shinagawa Gut Clinic, Tokyo 108-0074, Japan; 4 Department of Gastrointestinal Oncology, Osaka International Cancer Institute, Osaka 541-8567, Japan; 5 Department of Gastroenterological Chemotherapy, Cancer Institute Hospital, Japanese Foundation for Cancer Research, Tokyo 135-8550, Japan; kchin@jfcr.or.jp (K.C.); mariko.ogura@jfcr.or.jp (M.O.)

**Keywords:** esophageal fistula, esophageal cancer, self-expandable metallic stent, palliative treatment, dysphagia

## Abstract

Patients with malignant esophageal fistulas often experience dysphagia and infection, resulting in poor prognoses. Self-expandable metallic stent (SEMS) placement is a palliative treatment option; however, its efficacy and safety are unclear. We aimed to determine the efficacy and safety of SEMS placement for malignant esophageal fistulas. We retrospectively investigated patients who underwent SEMS placement for malignant esophageal fistulas between 2013 and 2022 at the Cancer Institute Hospital. Dysphagia scores (DSs) before and after SEMS placement, adverse events, and overall survival from SEMS placement until death were evaluated. A total of 17 patients underwent SEMS placement, including 12 and 5 patients with esophageal and lung cancers, respectively. Prior treatments included chemoradiotherapy (*n* = 11), radiotherapy (*n* = 4), and chemotherapy (*n* = 4); two patients underwent palliative radiotherapy after chemotherapy. All procedures were technically successful. After SEMS placement, 14 (82.4%) patients were able to consume semisolid or solid food (DS ≤ 2). Major adverse events were encountered in only one case. The median survival time after SEMS placement was 71 days (range 17–247 days). SEMS placement allowed most patients to resume oral intake with a low rate of major adverse events. SEMS placement is a reasonable palliative treatment option for patients with malignant fistulas who have poor prognoses.

## 1. Introduction

Most patients with esophageal cancer have advanced disease at the time of diagnosis because it does not show clinical symptoms in the early stages and progresses rapidly; therefore, it has a low survival rate of 10–30% in most countries [1,2,3,4]. In Japan, despite advancements in endoscopy and enhanced diagnostic capabilities leading to the early detection of esophageal cancer, 60% of all cases are still diagnosed as advanced cancers with poor prognoses [5]. Dysphagia due to malignant esophageal strictures, associated with weight loss and nutritional deficiency, is a common symptom in patients with advanced esophageal cancer. Treatment options for malignant esophageal strictures include surgery and chemoradiotherapy (CRT) if radical treatments are tolerable, and chemotherapy, radiotherapy (RT), and stent placement as palliative treatments. Indications for these treatments depend on the degree of disease progression and the patient’s general condition [6]. Among these, self-expandable metallic stent (SEMS) placement has been widely accepted as a palliative treatment for patients with incurable disease stages [7,8].

An esophageal fistula is one of the most critical complications of esophageal and lung cancers with mediastinal invasion or lymph node metastasis [9]. It can occur because of tumor invasion to adjacent organs or tissue damage related to therapeutic intervention, especially after CRT [9,10,11,12]. The incidence of esophageal fistula ranges from 10 to 22% among patients treated with CRT for unresectable esophageal squamous cell carcinoma (SCC) [13,14,15]. The diagnosis of an esophageal fistula is made mainly by CT but is also determined comprehensively via a combination of endoscopy, bronchoscopy, and contrast esophagography. Patients with esophageal fistulas typically manifest symptoms such as coughing after oral intake, aspiration, fever, dysphagia, and chest pain [16]. They have difficulty eating and drinking, resulting in nutritional failure. Furthermore, they have extremely poor prognoses owing to repeated transfistula infections [16]. Consequently, early detection and immediate therapeutic intervention are crucial. Unfortunately, as the majority of patients present with incurable stages at the diagnosis of fistulas, the fundamental approach is palliative treatment for the relief of dysphagia and the control of infections. Percutaneous gastrostomy and total parenteral nutrition using an implanted port catheter are treatment options; however, they cannot solve the problem of the inability to intake anything orally. Bypass surgery can be another option for patients with fistulas; however, the indicated patients are limited to those in an unstable condition because of its high invasiveness [17,18]; moreover, it is performed at limited institutions. Therefore, SEMS placement has been mainly preferred because it has the advantage of providing immediate relief from dysphagia and improving quality of life.

While SEMS placement has been widely used for esophageal stricture or fistula, it has sometimes resulted in life-threatening adverse events such as perforation, fistula formation, massive hemorrhage, and stent migration. Several researchers have investigated the efficacy and safety of SEMS placement and the risk factors of these adverse events [7,19,20,21,22]. However, the majority of studies have primarily included cases of malignant esophageal stricture. Patients with esophageal fistulas are often in poorer general condition than those with esophageal stenosis, obviously increasing the potential risk of the adverse events of airway obstruction and esophageal perforation. Moreover, as the application of SEMS placement is more complicated in patients with esophageal fistula, we have to consider the risk of airway obstruction, the possibility of simultaneous tracheal stenting, and the positional relationship of fistula and stenosis, so that SEMS can effectively seal the fistula. Thus, the outcomes of these patients must be investigated and their detailed various backgrounds should be obtained. Nevertheless, there have been few reports verifying the effectiveness and safety of SEMS placement for esophageal fistulas. Therefore, we retrospectively investigated the efficacy and safety of SEMS placement in patients with malignant esophageal fistulas.

## 2. Materials and Methods

### 2.1. Patients

We enrolled patients who underwent SEMS placement for malignant esophageal fistulas between November 2013 and September 2022 at the Cancer Institute Hospital. Clinical data were retrospectively collected from medical records. We considered SEMS placement in patients fulfilling the following criteria: (1) patients were diagnosed with unresectable malignant diseases by pathological examination; (2) radical treatments, such as surgery or CRT, were not indicated due to advanced disease stage and/or the patient’s general condition; (3) palliative RT was not scheduled after SEMS placement; and (4) the proximal margin of tumor growth was at least 2 cm below the pharyngoesophageal junction. We excluded patients for whom SEMS placement was deemed ineffective due to the absence of stenosis or a fistula on the oral side of the stenosis. Additionally, we refrained from SEMS insertion in patients with a high risk of airway obstruction due to the possibility of the trachea or bronchi being compressed by SEMS placement. It should be noted that tracheal stent insertion is not performed at our institution. If a fistula is confirmed involving the trachea via CT, a SEMS is not inserted because there is a high risk of airway obstruction. On the other hand, when a fistula is identified in the main bronchus, the decision regarding SEMS placement is made after discussion in a multidisciplinary conference with surgeons, oncologists, radiologists, and endoscopists in each case. However, in cases where a tumor apparently compresses the main bronchus, SEMS placement is withheld. When a fistula is observed in a peripheral bronchus rather than the main bronchus, we usually apply SEMS placement, considering them low-risk cases for airway obstruction. All procedures were performed in accordance with the principles of the Declaration of Helsinki. This study was approved by the Ethics Committee of the Institutional Review Board (No. 2022-GB-166), and the requirement for informed consent was waived because of the retrospective nature of the study.

### 2.2. Placement of SEMS

An esophageal fistula was diagnosed when a connection between the esophagus and the adjacent organs was detected using endoscopy, CT, and/or bronchoscopy. We performed endoscopies using a thin endoscope (GIF-XP290N, GIF-1200N; Olympus Medical System, Tokyo, Japan) to assess the length of the stenosis and the location of the fistula before SEMS placement. We mainly used Niti-S stents (partially covered or long-covered) (Taewoong Medical, Seoul, Korea). We selected a long-covered Niti-S stent with an anti-reflux valve when the distal edge of the stent was located near or in the stomach. If the tumor was located near the esophageal orifice, we selected a fully covered HANAROSTENT for the upper esophagus with a short oral side flare (M.I. Tech, Seoul, Korea). The stent length was chosen so that it covered at least 2 cm beyond each tumor edge, particularly on the oral side, covering the fistula in the center of the stent. All SEMS placements were performed under fluoroscopic guidance, as previously described [23]. All patients were sedated with midazolam and pethidine hydrochloride during the procedure. Briefly, we identified the stenosis and fistula using a thin endoscope, and indicated the tumor margin using an external marker on the body surface as confirmed using fluoroscopy. Then, we inserted a guidewire into the stomach. After that, a delivery system was inserted over the guidewire to release the SEMS under fluoroscopic guidance. Finally, stent position and patency were confirmed endoscopically. We performed esophagography 3 days after SEMS placement to confirm the absence of leakage and allowed the patients to initiate oral intake of liquid or semisolid food.

### 2.3. Definitions

Performance status was defined according to the Eastern Cooperative Oncology Group (ECOG) criteria [24]. The location in esophagus was classified in accordance with the Japanese Classification of Esophageal Cancer edited by the Japan Esophageal Society [25]. Dysphagia scores (DSs) were evaluated before SEMS placement and at discharge as follows: 0, able to eat a normal diet; 1, unable to swallow certain solids; 2, able to swallow semisolid foods; 3, able to swallow liquids only; and, 4, unable to swallow anything [26]. Technical success was defined as proper positioning and deployment of the stent at the site of stenosis and fistula. Major adverse events were defined as life-threatening, such as perforation, fistula formation, and massive hemorrhage, as described previously [23]. Moderate adverse events were defined as events that were not life-threatening but required intervention, including migration and restenosis (overgrowth/ingrowth of tumor). Minor adverse events were defined as transient events that could be resolved within a few days, such as chest pain and fever. Overall survival was calculated from the day of SEMS placement to the patient’s death using the Kaplan–Meier method.

## 3. Results

### 3.1. Patient Characteristics

Patient characteristics are outlined in Table 1. During the study period, 17 patients with malignant esophageal fistulas were treated with SEMS placement. In all cases, the thin endoscope could be passed through the stricture before SEMS placement. The fistula was caused by esophageal SCC in 12 patients and by lung cancer in 5 patients (1 with SCC and 4 with adenocarcinoma). There were no cases of fistulas caused by prior SEMS placement. Of the 17 patients, 14 (82.4%) were men, and the median age was 67 years (range 42–89 years). The most common location of fistula was in the middle thoracic esophagus (70.6%). The median length of stenosis was 80 mm (range 20–130 mm). Fistula formation was observed in the bronchi (*n* = 9), lungs (*n* = 4), mediastinum (*n* = 3), and metastatic lymph nodes (*n* = 1). Prior treatments included CRT (*n* = 11), RT (*n* = 4), and chemotherapy (*n* = 4) (including two patients with lung cancer who underwent palliative RT after chemotherapy). The median time from the first diagnosis of esophageal SCC or lung cancer to SEMS placement was 175 days (range 50–3228 days). The median dose of radiation in the 15 patients who underwent RT was 60 Gy (range 40–64 Gy). The median time from the completion of radiation to SEMS placement was 81 days (range 14–1674 days). Two patients underwent aortic stent graft placement for esophagoaortic fistula before esophageal SEMS placement.

### 3.2. Efficacy

**Figure 1:**
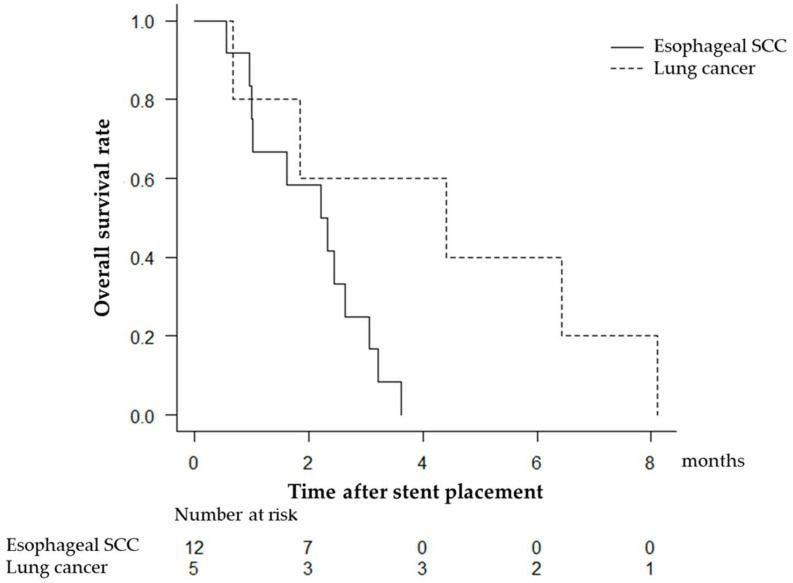
Overall survival after SEMS placement was determined using the Kaplan–Meier method. Kaplan–Meier curves are presented separately because the prognosis of each cancer is different. SCC: squamous cell carcinoma.

All SEMS placement procedures were technically successful. There were no cases that needed balloon dilation before SEMS placement. We used a partially covered Niti-S stent, a long-covered Niti-S stent, and a HANAROSTENT in 13, 3, and 1 patients, respectively (Table 2). Of the 12 patients who could not swallow liquids (DS 4) before SEMS placement, 1 and 9 patients were able to swallow some solid foods (DS 1) and semisolid foods (DS 2), respectively. Regarding the location, the proportions of patients who achieved DS ≤ 2 after SEMS placement were 60% for the upper thoracic esophagus and 91.7% for the middle thoracic esophagus. One patient was unable to start oral intake because of no improvement in pre-existing pneumonia and pleural fluid. The median time to resume oral intake was 4 days (range 1–13 days). Five (29.4%) patients (three patients with esophageal SCC and two patients with lung cancer) underwent chemotherapy after SEMS placement. All the patients died before data collection. The median survival time after SEMS placement was 71 days (range 17–247 days), and six (35.3%) patients survived for more than 3 months. Overall survival after SEMS placement is shown in Figure 1. The median survival times were 2.3 months for esophageal SCC and 4.4 months for lung cancer.

### 3.3. Adverse Events

Only one major adverse event, an esophagorespiratory fistula, occurred in a patient with esophageal SCC who had an SEMS inserted in the upper thoracic esophagus (Table 3). The fistula was confirmed at the proximal edge of the SEMS 55 days after placement. It was difficult to efficiently cover the fistula using the SEMS because the fistula was located at the proximal edge of the esophageal SCC, and the patient died 55 days after the detection of the fistula. Moderate adverse events, such as migration and restenosis (overgrowth/ingrowth of tumor), did not occur in any patient. The most common minor adverse events were chest pain (6, 35.3%) and fever (5, 29.4%). Two (11.8%) patients had pre-existing pneumonia, and pleural effusion worsened after SEMS placement. One patient continued antibiotic therapy and started oral intake, whereas the other could not initiate liquid intake and died 29 days after SEMS placement.

## 4. Discussion

Patients with esophageal fistulas are often recommended to discontinue oral intake because of the risk of respiratory or mediastinal infections from the fistula, resulting in nutritional deficiency and poor prognosis. However, in our study, after SEMS placement, 82.4% of patients were able to consume semi-solid food or some solid foods (DS ≤ 2). We demonstrated the efficacy of SEMS placement for esophageal fistulas in palliative treatment for relief from dysphagia in most patients in this study.

Regarding the location of SEMS, 91.7% of patients with SEMS in the middle thoracic esophagus achieved DS ≤ 2 after SEMS placement; one patient could not try oral intake due to worsened pneumonia. In contrast, in patients with SEMS in the upper thoracic esophagus, only 60% could ingest solid or semisolid food (DS ≤ 2). This was because the stricture was closer to the esophageal orifice in patients with SEMS in the upper thoracic esophagus, resulting in smaller food-pooling spaces even after SEMS placement.

The incidence of major adverse events was limited to only one case of stent-induced fistula (5.9%), where the SEMS was inserted in the upper thoracic esophagus. Previous studies on SEMS placement for malignant esophageal fistulas have reported that the incidences of massive hemorrhage and fistula/perforation were 4.3–42% [20,27,28] and 0–4.3% [20,27,28], respectively. Massive hemorrhage occurred most frequently among patients with major adverse events in each report, although we did not observe massive hemorrhage in this cohort. The concomitant insertion of tracheal stents has been reported as a predictor of massive hemorrhage [20,29]. The reason for massive bleeding is thought to be an increase of mechanical pressure on the adjacent tissues, ultimately leading to tissue necrosis. Our study did not include patients requiring tracheal stents, which could be one of the reasons for the absence of massive hemorrhage. In addition, the thin endoscope could be passed through the stricture before SEMS placement in all patients in our cohort. Of course, we would place a SEMS in a fistula case with stenosis which the thin scope could not pass, as we usually perform in cases with simple stenosis; however, we did not encounter a complete occlusion case with a fistula. Because all our cases were diagnosed with fistula in the treatment period of cancer, we were able to detect fistula earlier with regular CT scans and hospital visits. For another reason, we sometimes pushed scopes a little more if required and manipulated scope angles, trying hard to pass through, because it is better to understand the whole positional relationship of stenosis and fistula. The absence of complete occlusion cases could have contributed to the low frequency of major adverse events such as perforation, and the SEMS placement would carry higher risks in such cases.

The median survival time after SEMS placement was 71 days (range 17–247 days), which we think is a good outcome as previously long treated advanced cancer cases, and it is similar to that reported in previous studies (72–111 days) [22,27,28,30]. One patient who developed a fistula died 110 days after SEMS placement, which was not shorter compared with outcomes for other patients. This is similar to the outcomes of our previous study on SEMS placement for esophageal stricture/fistula, where the median survival time after the adverse events of perforation/fistula was 55 days (range 7–238 days), which was not so short in spite of severe adverse events [23]. On the contrary, massive bleeding following SEMS placement is fatal and leads to death within 24 h [20,29], whereas the survival time after fistula formation is not always brief. This may be because we can avoid secondary severe events by reinsertion of a SEMS or discontinuation of oral intake, consequently resulting in partial control of the transfistula infection.

It has been reported that SEMS placement in patients with prior RT/CRT carries a higher risk of major adverse events [19,21,31,32]. We previously reported that the incidence of major adverse events following SEMS placement with a Niti-S stent was sufficiently low, even among patients with a history of RT/CRT [23]. Similarly, some researchers have indicated that the Niti-S stent decreases the risk of major adverse events [7,33]. Niti-S stents have a low-radial force [34], resulting in less stress on the esophageal wall. In this study, although 88.2% of the patients had received prior RT/CRT, the incidence of major adverse events was low. This might be attributed to the predominant use of low-radial force stents.

The most frequent adverse events were minor, such as chest pain and fever, and these could be managed with symptomatic therapies. The reason for the low frequency of moderate adverse events (migration and restenosis) compared with that in previous reports [27,28] may be due to the small number of patients. The placement of SEMS across the gastroesophageal junction can be the cause of gastroesophageal reflux; however, this problem has been preventable with the use of stents with antireflux mechanisms [35]. In this study, a long-covered Niti-S stent with an antireflux mechanism was used in three patients who had fistulas in the middle thoracic esophagus and who also had tumor invasion into the lower thoracic esophagus. Including these three cases, there was no case of gastroesophageal reflux in our study, although there exist limitations in detecting patients’ symptoms in detail due to the retrospective nature of a medical records review.

Regarding patients who developed esophageal fistulas at diagnosis and required radical treatment, the efficacy of CRT for fistula closure was 91.7% [36]. On the other hand, in cases where radical treatment is not indicated, SEMS placement is the most common option, while RT and bypass surgery are also considered palliative treatment options. There have only been a few reports regarding RT for esophageal fistulas. Patients treated with RT had prolonged survival compared with those receiving supportive care [16], and RT achieved fistula closure in 42.9% of patients [37]. While RT can be effective in some cases, it may take longer to have an effect than SEMS placement. Additionally, RT is often not applicable to patients with fistulas because many of them have already undergone RT/CRT. Bypass surgery cannot be performed in all patients with fistulas because it is a much more invasive procedure with a high incidence of severe complications, such as anastomotic leakage [17], especially in patients with a history of prior RT/CRT. Because the prognosis of patients with fistulas is usually poor, palliative treatment should be minimally invasive and immediately effective. Thus, SEMS placement is a good option in many cases. In contrast, RT and bypass surgery should be considered for patients with a better prognosis or for those for whom SEMS placement is not suitable because of the absence of stenosis or the presence of a fistula at the oral side of the stenosis.

Patients with fistulas are more likely to have a poor general condition because of the underlying disease and repeated episodes of infection, especially pneumonia. Pulmonary infection at the time of SEMS placement was associated with poor prognosis [28]. In this study, pneumonia worsened in two patients after SEMS placement. One patient was anxious about the progression of pneumonia and hesitated to initiate oral intake. However, pneumonia and pleural effusion did not improve, and the patient died 29 days after SEMS placement without even ingesting water. Another patient with pneumonia initiated oral intake after the absence of leakage was confirmed by esophagography. Because patients with fistulas usually have limited survival, oral intake is worth considering, even in patients with severe pneumonia, to improve their quality of life.

This study had several limitations: (1) this was a single-center retrospective study with a small number of patients; (2) long-term adverse events after SEMS placement could not be followed in detail because of the retrospective nature of this study; (3) the incidence of major adverse events may have been influenced by the aspect that this study did not include more serious cases, such as those requiring concomitant tracheal stents or those with severe stenosis, which does not allow even a thin endoscope to pass through; (4) there was no control group comprising patients without fistulas; and (5) since the decision to perform SEMS placement was left at the discretion of the physician, it is possible that patients with severe general conditions were not included in this study. A prospective, multicenter trial with a large number of patients is required for further investigations.

## 5. Conclusions

In conclusion, SEMS placement for malignant esophageal fistulas was technically feasible, and most patients were able to resume oral intake with a low rate of major adverse events. SEMS placement is a reasonable palliative treatment option for patients with malignant esophageal fistulas and poor prognoses.

## Figures and Tables

**Table 1:** Patient characteristics.

	Total	Esophageal SCC	Lung Cancer
	*n* = 17	*n* = 12	*n* = 5
Age (years), median (range)	67 (42–89)	67.5 (51–89)	59 (42–71)
Sex (M), *n* (%)	14 (82.4)	10 (83.3)	4 (80.0)
Performance status, n			
0/1/2/3/4	0/7/7/3/0	0/5/5/2/0	0/2/2/1/0
Location, *n* (%)			
Ut	5 (29.4)	4 (33.3)	1 (20.0)
Mt	12 (70.6)	8 (66.7)	4 (80.0)
Lt, Ae	0 (0.0)	0 (0.0)	0 (0.0)
Stenosis length (mm), median (range)	80 (20–130)	85 (20–130)	60 (40–80)
Fistula formation, *n* (%)			
Bronchus	9 (52.9)	6 (50.0)	3 (60.0)
Lung	4 (23.5)	3 (25.0)	1 (20.0)
Mediastinum	3 (17.6)	3 (25.0)	0 (0.0)
Lymph node	1 (5.9)	0 (0.0)	1 (20.0)
Anticancer treatment before SEMS placement *, *n* (%)			
CRT	11 (64.7)	8 (66.7)	3 (60.0)
RT	4 (23.5)	2 (16.7)	2 (40.0)
Chemotherapy	4 (23.5)	2 (16.7)	2 (40.0)
Dose of RT (Gy), median (range)	60 (40–64)	60 (40–60)	60 (45–64)
Interval between the first diagnosis and SEMS placement (days), median (range)	175 (50–3228)	158 (50–644)	813 (358–3228)
Interval between the completion of RT and SEMS placement (days), median (range)	81(14–1674)	65.5 (14–459)	233 (28–1674)
Aortic stent graft, *n* (%)	2 (11.8)	2 (16.7)	0 (0.0)

n: number, SCC: squamous cell carcinoma, Ut: upper thoracic esophagus, Mt: middle thoracic esophagus, Lt: lower thoracic esophagus, Ae: abdominal esophagus, SEMS: self-expandable metallic stent, CRT: chemoradiotherapy, RT: radiotherapy; * Two patients with lung cancer underwent palliative RT after chemotherapy.

**Table 2:** Efficacy of SEMS placement.

	Total
	*n* = 17
Technical success, *n* (%)	17 (100)
Procedure time (min), median (range)	15 (5–30)
Predilated with a balloon, *n* (%)	0 (0.0)
SEMS, *n* (%)	
Niti-S partially covered stent	13 (76.5)
Niti-S long-covered stent	3 (17.6)
HANAROSTENT	1 (5.9)
Stent length (cm) (8/10/12/15)	1/1/4/11
DS before SEMS placement, *n*	
0/1/2/3/4	0/0/3/2/12
DS after SEMS placement, *n*	
0/1/2/3/4	0/2/12/2/1
Successful resumption of oral intake (DS ≤ 2), *n* (%)	14 (82.4)
Unable to swallow liquids after SEMS placement (DS 4), *n* (%)	1 (5.9)
Time to resume oral intake (days), median (range)	4 (1–13)
Survival time after SEMS placement (days), median (range)	71 (17–247)
Subsequent anti-cancer treatment after SEMS placement, *n* (%)	
Chemotherapy	5 (29.4)

*n*: number, DS: dysphagia score, SEMS: self-expandable metallic stent.

**Table 3:** Adverse events following SEMS placement.

	Total
	*n* = 17
Major adverse events, *n* (%)	
Fistula	1 (5.9)
Perforation	0 (0.0)
Hemorrhage	0 (0.0)
Moderate adverse events, *n* (%)	
Migration	0 (0.0)
Restenosis(overgrowth/ingrowth of tumor)	0 (0.0)
Minor adverse events, *n* (%)	
Chest pain	6 (35.3)
Fever	5 (29.4)

*n*: number, SEMS: self-expandable metallic stent.

## Data Availability

All the data from this study are available from the corresponding author upon request.
